# Foot and Soccer Referees’: A Pilot Study Searching “Performance” Throughout Prevention

**DOI:** 10.3389/fphys.2018.01009

**Published:** 2018-07-25

**Authors:** Luigi Gianturco, Bruno D. Bodini, Vincenzo Gianturco, Fabrizio E. Pregliasco, Marta Cascio, Antonio Serafin, Maurizio Turiel

**Affiliations:** ^1^Cardiology Unit, IRCCS Galeazzi Orthopedic Institute, Milan, Italy; ^2^Rehabilitation Unit, IRCCS Galeazzi Orthopedic Institute, Milan, Italy; ^3^Fondazione Filippo Turati Onlus, Rome, Italy; ^4^Department of Biomedical Sciences for Health, IRCCS Galeazzi Orthopedic Institute, University of Milan, Milan, Italy; ^5^Podiatry Service, IRCCS Galeazzi Orthopedic Institute, University of Milan, Milan, Italy

**Keywords:** soccer referees, foot, performance, Yo-Yo test, speckle-tracking based strain imaging

## Abstract

Soccer refereeing is a “not-conventional” sport in which aerobic workload is prevalent. Along the years, several studies have attempted to define best markers of referees’ performance. Many studies focused their attention on field tests and their relationship with aerobic power. Instead, in this study, starting by a medical assessment satisfying the FIFA 11+ criteria for injuries prevention, we have investigated the foot of soccer referees and we have also wanted to find possible and/or unexpected improvements in performance. As performance marker, we have used the referral field test for soccer referees that is internationally validated and known as Yo-Yo test (YYiR1). While standardized foot posture index (FPI) questionnaire was used for screening foot referees conditions (40 young, all men by sex, with mean age 23.47 ± 4.36). Analyzing collected data, we have demonstrated by means of Read–Cressie Chi square test that neutral FPI is an important favor item affecting YYiR1 results. Further studies will be necessary in order to confirm our pilot investigation.

## Introduction

The international governing body of soccer, well-known as FIFA, has been launching since 2014 the 11+ program for promoting health and prevention throughout football (and/or soccer) playing. The 11+ try to prevent injuries, especially for foot and ankle (Barengo et al., 2014). In literature, we can found several studies about foot and/or ankle in soccer players (Cain et al., 2007; Sadigursky et al., 2017) but there is lack of sufficient evidence for referees, generally studied for other determinants (Castagna et al., 2007; Weston et al., 2012).

Moreover, it is also known that foot posture might influence the function of limb and so play a role in predisposition to overuse injuries (Burns et al., 2005). Regarding injuries’ management, FIFA takes much care on pre-competition medical assessments (PCMA) and also referees are more and more studied (Bizzini et al., 2012).

Indeed, as above cited, soccer referees have been extensively studied for “performance” along the years. So, most of studies have focused their attention on aerobic indexes such as VO2max (Castagna and D’ottavio, 2001; Gianturco et al., 2017) because of refereeing mainly involves aerobic metabolism (Casajus and Castagna, 2007).

**FIGURE 1:**
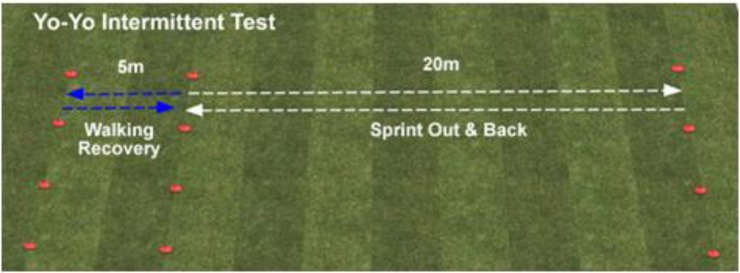
Yo-Yo test graphic explanation.

However, many factors may influence the performance of the so-called 23rd player (Reilly and Gregson, 2006) and the growing financial importance of soccer competitions requires higher prestations ensuring with best levels of physical fitness (Mallo et al., 2009). During a standard match, a referee covers about 10–12 km (Krustrup and Bangsbo, 2008) and steadiness of physical output is pivotal to make right decisions (Weston et al., 2012). So, leading national and international federations periodically assess physical integrity and also performance of referees by means of specific fitness tests (Castagna et al., 2007). Currently, the most common used tool to assay soccer referees’ capacities is Yo-Yo intermittent recovery level 1 test (YYiR1) designed for the first time by Bangsbo in 1994 (Bangsbo, 1994). This field test has completely substituted 12-min run test (12MRT), usually well-known as Cooper test, which was previously used by most important referees’ boards (Castagna et al., 2005). We can see Yo-Yo test graphic explanation in **Figure 1**.

Moreover, governing boards generally evaluate referees’ physical fitness during the competitive season with the specific contribution of a biomedical staff. By working together professional figures (medicine doctors, physiotherapists, and athletic trainers) continuously search novel ways to improve physical outcomes.

So, we can find several studies inhering training and cardiorespiratory responses but no study has focused the attention on foot’s role.

In order to define foot of soccer referees, we have chosen foot posture index (FPI) which may better reflect foot’s condition than other more complex markers (Buldt et al., 2013) whenever feasible. In fact, other measurements can be bulky in daily practice and more susceptible to errors compared with FPI. We also know that FPI is influenced by age and documented systemic (i.e., diabetes, systemic inflammatory diseases) and/or peripheral pathologies (Redmond et al., 2008). But our population was not aging and it was without any history of foot’s affection.

Therefore, the aim of this study was to collect data on foot’s referees and secondarily analyze the impact of foot’s determinants in YYiR1 findings.

## Materials and Methods

### Participants

The study was carried out examining 40 male soccer referees (mean age 23.47 ± 4.36, BMI 20.10 ± 1.10) belonging to Italian Soccer Referees Association (AIA). The officials who volunteered for the present research possessed median of 5 years of refereeing experience and all the referees trained at least three times a week, following a supervised global training program. The protocol was performed in accordance with the Declaration of Helsinki (Millum et al., 2013) and met the ethical standards in Sport and Exercise Science Research.

Local internal Ethics Committee of AIA has approved the study and all participants signed written consent. The study did not provide any pharmacological assessment and/or invasive procedures.

### Anthropometrical and Instrumental Measurements

Subjects enrolled underwent a standard cardiac examination and then an echocardiographic evaluation. Echo assessments were all performed by only one expert cardiologist with specific competence in studying of athletes. For all of exams was used a commercial ultrasound machine (Epiq 7; Philips Medical System, Andover, MA, United States) equipped with a conventional probe. 2D measurement of left ventricle (LV) cavity diameters, wall thickness, and mass was assessed according to American Society Echocardiography criteria (Lang et al., 2005). LV ejection fraction (EF) was measured with the biplane Simpson’s rule from the apical four- and two-chamber views, while LV mass was measured with Devereux’s formula (Devereux and Reichek, 1977), and finally analysis of 2D strain imaging was performed offline with commercially available software (QLAB version 10.0; Philips Medical Systems, United States).

This latest version was developed following the recommendation of a joint American Society of Echocardiography and American Heart Association document, aimed to improve standardization of the strain analysis and reduce intervendor variability (Geyer et al., 2010).

Apical four-chamber views were acquired achieving a high frame rate (70–80 frames/s) and fitting the entire LV in the echocardiographic plane (Galderisi et al., 2015). Attention was paid to acquire cardiac cycles of the same standardized length and during the same respiratory phase (expiration); so, three cardiac cycles were stored in cine-loop format for offline analysis in order to assess end-systolic LV longitudinal strain. The endocardial border was traced on an end-diastolic frame and subsequently automatically tracked; the tracking was verified in real time and corrected by adjusting the region of interest or manually correcting the border. End-systole was identified as corresponding to aortic valve closure as measured by pulsed-Doppler.

The software represents myocardial deformation in the form of time–strain graphs in which it is possible to identify the different phases of the cardiac cycle as follows: a negative wave is observed during systole, which reaches its negative peak at the time of aortic valve closure and represents maximal longitudinal myocardial shortening during contraction; during diastole, the strain values progressively increase toward the original length.

The time–strain curves were obtained and analyzed by two independent observers who were blinded to the clinical data. Inter-observer variability was calculated using the Bland–Altman (Bland and Altman, 1986) method to compare the measurements made by the two observers in 10 randomly selected subjects, and was <5% for global longitudinal strain (GLS).

### Yo-Yo Test

The YYiR1 (also known briefly as Yo-Yo test) is the current most valuable tool to measure specific fitness in soccer referees. It consists of 2 × 20 m runs back and forth between two lines at a progressively increasing speed controlled by audio signals. When the referees twice failed to reach the corresponding line in time, the test finished and the distance covered was recorded as personal result. Minimum level of coverage is normally set for Italian referees at 2800 m.

Each testing session was performed with well-rested subjects as already done in the recent past (Fanchini et al., 2015). More in detail, referees have kept up moderate exercise during 24–48 h preceding YYiR1 (i.e., 20–30 min running at 70% of individual maximal heart rate). Referees performed test on natural turf because of it was the prevalent type of pitch on which they “worked.” Finally, the environmental conditions during tests were similar for all participants because of it was executed in the same single day.

### Foot Posture Index

The FPI is a questionnaire quantifying foot posture on the basis of six criteria (Redmond et al., 2008). All of subjects were screened by means of a standard physical examination of feet followed by the FPI questionnaire that have collected whole data. These evaluations were performed by a unique podiatric specialist in all subjects in the same seasonal pre-competitive period. Then, collected data were inserted in a database for statistical computations. The reliability of acquisitions was assessed by means of *test–retest* strategy and a mean Pearson correlation coefficient >0.81 was obtained (Shieh, 2010). Compare measurements made by the only observer were at two very close points in time.

### Statistical Analysis

Continuous data were expressed as mean ± standard deviation (*SD*) while categorical data as frequencies.

Non-parametric Chi Square test (Read–Cressie method) (Cressie and Read, 1989) was used to identify the supposed negative influence of abnormal FPI in YYiR1 performance. The alfa level of statistical significance was set at 5% (*p* ≤ 0.05) *a priori*.

The free statistics software package R was used for computations (R Development Core Team, 2008).

## Results

The basal characteristics of enrolled subjects in this study are shown in **Tables 1**, **2**, respectively. The mean age reveals a young population without abnormalities inhering weight and especially body mass index (BMI). Summarizing echocardiophic results, cardiac chambers were normal for diameters and indexed mass of LV. Systolic function of LV was regular with not abnormal values for EF and GLS. We observed any valvular disease and also right chambers were normal for dimensions and function (i.e., normality for conventional indexes TAPSE and PAPs).

**Table 1:** Basic characteristics of population.

Parameters	Means (±*SD*) or %
Age (years)	23.47 (4.36)
Height (m)	1.78 (0.12)
Weight (Kg)	74.20 (5.61)
BMI (Kg^∗^m^−2^)	20.10 (1.10)
SBP (mmHg)	123.51 (8.88)
DBP (mmHg)	75.42 (6.16)
HR (beats^∗^min^−1^)	62.32 (7.04)
FPI neutral (%)	71.5
NTS/year	148.50 (2.10)
YYiR1 distance coverage (m)	2800 (110)

*BMI, body mass index; SBP, systolic blood pressure; DBP, diastolic blood pressure; HR, heart rate; FPI, foot posture index; NTS, total number of traning sessions per year; YYiR1, Yo-Yo test. Continous data are presented as means (± SD) while categorical data were expressed in percentage (%).*

**Table 2:** Echocardiographic parameters.

Parameters	Means (±*SD*)
LVEDD (mm)	50.44 (4.16)
LVESD (mm)	31.71 (3.33)
LVEDV (ml)	112.22 (3.84)
LVESV (ml)	41.38 (3.22)
EF (%)	62.12 (4.76)
LV indexed mass (g^∗^m^−2^)	104.26 (3.32)
GLS (%)	22.14 (2.54)

*LVEDD, left ventricular end-diastolic diameter; LVESD, left ventricular end-systolic diameter; LVEDV, left ventricular end-diastolic volume; LVESV, left ventricular end-systolic volume; EF, ejection fraction; LV indexed mass, left ventricle indexed mass; GLS, global longitudinal strain, expressed as usual as negative value. Data are presented as means (±SD).*

Finally, in **Table 3**, there are shown findings of inferential statistics process. As Chi square test (performing by means of Read–Cressie method variant) revealed, there was an important relationship between foot and YYiR1 performances (*p* < 0.001). In other words, neutral foot positively “affects” YYiR1 results.

**Table 3:** Chi square test (Read–Cressie method).

FPI neutral	YYiR1 > 2800 m		Total
	Yes	Not	
Yes	33	2	35
Not	1	4	5
Total	34	6	40

*P < 0.001; Chi square value = 16.2073; degrees of freedom = 1. FPI, foot posture index; YYiR1, YoYo intermittent recovery test (type 1: classic).*

## Discussion

As written in Section “Introduction,” there are not any studies about foot and refereeing and relatively few data are available about FIFA PCMA in soccer referees (Bizzini et al., 2012). We have found some manuscripts analyzing foot on indoor football (Cain et al., 2007) and/or about handball and football players, but nothing about referees (Hagen et al., 2016). We also knew that in older ages it is common to find foot disorders (as indicated in the so-called Framingham Foot Study; Hagedorn et al., 2013) but our sample was younger. Besides that, we have investigated sports men without heart diseases but also with the absence of known musculoskeletal abnormalities.

We did not enroll women because of the majority of soccer referees are men. So, we had a omogeneous group on the basis of sex, age, and BMI and such parameters did not affect our statistics computations.

Moreover, for FIFA Medical Department, prevention programs such as FIFA 11+ and/or PCMA in general are more and more crucial to use science in soccer and for soccer. So, with our study, we have primarily obtained a data collection useful for guiding specialists in referees’ physical management and programming future studies.

Focusing the attention on results, we observed: ECG tracings ok such as echo assessments. Similarly, the number of training sessions per year (NTS/y) did not have a great importance in statistical analysis because of their not wide amplitude. The goodness of this item is unanswerable because NTS/y were collected by athletic trainers of AIA by means of institutional dedicated web platform day by day with any possibility of delayed modifications.

In Section “Introduction,” we have just clarified the role of field tests to essay physical fitness of soccer referees, and moreover, we have underlined the pivotal use of aerobic measurements. So, YYiR1 is actually the best “performer” to do that and it was used as main marker also by us. Obviously, there is an indirect tool to assess aerobic power but as already demonstrated by Bangsbo et al. (2008) there is a direct relationship between YYiR1 findings and maximum oxygen consumption (VO2max) known by scientific community as the more accurate method to measure aerobic power. So, the best minor invasive choice to assess aerobic performances was YYiR1. Besides that, field test as YYiR1 is a more reproducible tool for assessing aerobic power and then we have chosen it for our study.

Performing non-parametric statistics, we have demonstrated that the main influencer of YYiR1 was FPI. FPI is a standardized questionnaire used for revealing macroscopic foot’s disorders. A neutral score in each one of the six investigated items is suggestive of a normal feature, while positive and negative scores mean abnormal setting. We use FPI-6 items similar to FPI-8 items but better for standardized use in screening studies, as revealed by Evans et al. (2003) and Keenan et al. (2007). Despite some dynamic limitations, FPI is the most frequently reported measure of foot basilar characteristic and so it is a reasonable marker of function. Dynamic foot’s evaluations are more difficult to pursuit and might generate any reliable conclusions (Buldt et al., 2013).

As just indicated, in our population, FPI and YYiR1 were strictly related: so, we may softly affirm that in our group substantially omogeneous on the basis of age, BMI, and training grade one conceivable further strategy to improve field performance seems to be the “care of foot.”

In other words, foot conditions may significantly affect field tests as YYiR1 even if that assumption could also be considered self-evident.

We may conclude that referee of the future will move himself from standard training program to evoluted form of physical performance that might take advantage also of the care of foot in addition to classic strategies of fitness improvement and in addition to the study of foot exclusively for preventive objectives (Yeung and Yeung, 2001). Here it is the interesting challenge to carry on starting tomorrow.

## Conclusion

Our results may open new scenarios for biomedical AIA staffs in order to optimize foot outcomes and reach best overall results in soccer refereeing.

In recent past, studies regarding soccer referees’ world analyzed almost exclusively energy expenditure (Ardigò et al., 2015) and in order to measure that, the authors have chosen strategies such as match analysis (Ardigò, 2010). Starting today, we could also hypothesize a comprehensive foot study before starting competitions’ season using a specific and dedicated staff doing that (i.e., podiatrics collaborating with already existing biomedical teams).

However, further studies with more amplitude are necessary in order to clarify this pilot assessment and trace methodology of next works. For example, it may consider cardiopulmonary (CPX) assessment for evaluating maximum aerobic power and understanding its relationship and/or influence on echocardiographic and foot’s parameters analyzed in this paper.

Honestly, our data are quite limited to draw definitive conclusions and then future research should establish whether the foot is really one of the most items affecting referees’ performance.

About topics not sufficiently addressed yet, here they are some suggestions for future studies:

1. use of other more complex tools for assessing foot not only in its posture but also in dynamic scenarios
2. quantification of observed differences by age (as seen by Redmond et al.)
3. evaluation of hypothesized differences between “pitch” referees and assistant referees
4. different findings in relation with different turf (natural, like in this study or artificial).

## Author Contributions

LG drafted the manuscript. MC and AS helped to draft the manuscript and researched the literature. VG and BB took care of biostatistics contribution and besides that, BB gave contribution for English language. MT, AS, and FP commented on and participated in critical editing of this manuscript. All authors read and approved the final submission of text.

## Conflict of Interest Statement

The authors declare that the research was conducted in the absence of any commercial or financial relationships that could be construed as a potential conflict of interest.
